# Matrix-assisted laser desorption/ionization-time of flight MS as a tool for clonal discrimination in Streptococcus dysgalactiae subsp. equisimilis

**DOI:** 10.1099/jmm.0.002080

**Published:** 2025-09-29

**Authors:** Isis Hazelman Vieira dos Anjos, Caroline Lopes Martini, Luis Guilherme de Araujo Longo, Úrsula Santos Lopez, Tatiana de Castro Abreu Pinto, Agnes Marie Sá Figueiredo, Bernadete Teixeira Ferreira-Carvalho

**Affiliations:** 1 Departamento de Microbiologia Médica, Universidade Federal do Rio de Janeiro, Rio de Janeiro, RJ, Brazil; 2 Faculdade de Medicina, Instituto de Educação Médica (IDOMED), Universidade Estácio de Sá, Rio de Janeiro, RJ, Brazil; 3 Programa de Pós-graduação em Patologia, Faculdade de Medicina, Universidade Federal Fluminense, Niterói, RJ, Brazil

**Keywords:** biomarkers, clonality, matrix-assisted laser desorption/ionization-time of flight (MALDI-TOF), *Streptococcus dysgalactiae* subs. *equisimilis* (SDSE)

## Abstract

**Introduction.** *Streptococcus dysgalactiae* subsp. *equisimilis* (SDSE) is an emerging pathogen closely related to *Streptococcus pyogenes*, causing infections from mild to severe, including necrotizing fasciitis and toxic shock syndrome. Understanding bacterial diversity is crucial for monitoring the spread of antimicrobial-resistant and highly virulent strains. Clonal analysis by whole-genome sequencing (WGS) is costly, time-consuming and requires specialized bioinformatics expertise. PFGE-defined clones are often linked to human infections, but PFGE is also demanding and costly and results can vary between laboratories.

**Aim.** This study explores the use of matrix-assisted laser desorption/ionization-time of flight MS (MALDI-TOF MS) to detect discriminatory biomarkers that discriminate SDSE strains exhibiting distinct PFGE clonal types.

**Methodology.** MALDI-TOF MS spectra were generated from SDSE strains using a Microflex LT mass spectrometer (Bruker) and analysed with BioNumerics software v7.6. To validate the genetic relevance of PFGE pulsotypes, WGS and phylogenomic reconstruction were performed.

**Results.** Unique MALDI-TOF MS biomarker peaks consistently differentiated SDSE strains corresponding to PFGE patterns A and B, providing robust molecular signatures for discriminating these clonal types. Phylogenomic analyses further supported this distinction by clustering PFGE A and B strains into two distinct main clades with an elevated accuracy of 95.2 (95% confidence interval: 76.2–99.9%).

**Conclusion.** MALDI-TOF MS is effective not only for species identification but also for rapid and reliable assessment of SDSE clonal diversity. This approach has the potential to enable epidemiological tracking of specific clones, enhance understanding of SDSE in human infections and provide a practical tool for research and clinical surveillance.

**Impact Statement**

*Streptococcus dysgalactiae* subsp. *equisimilis* (SDSE) causes diseases ranging from mild illnesses to severe conditions, including necrotizing fasciitis and toxic shock syndrome. Tracking the diversity of SDSE strains is essential to understand their spread and development of antimicrobial resistance. Methods such as whole-genome sequencing or PFGE are effective but costly, time-consuming and require specialized expertise. This study evaluated the ability of matrix-assisted laser desorption/ionization-time of flight MS (MALDI-TOF MS) – a faster and more affordable method routinely used in clinical microbiology laboratories for bacterial identification – to discriminate SDSE strains based on unique biomarker profiles. We found that MALDI-TOF MS reliably identified specific biomarkers that differentiated SDSE strains into distinct PFGE groups. This approach provides a simpler and faster way to study bacterial diversity and track the spread of different strains in human infections. By making strain identification more accessible and efficient, MALDI-TOF MS has the potential to improve infection control, enhance outbreak monitoring and guide future research. These findings highlight the potential of this tool to address public health challenges posed by SDSE and other emerging pathogens, shaping the way bacterial infections are studied and managed.

## Data Summary

The authors confirm that all supporting data, code and protocols are provided within the article or in the supplementary data files.

## Introduction

The species *Streptococcus dysgalactiae* comprises two subspecies: *Streptococcus dysgalactiae* subsp. *equisimilis* (SDSE) and *Streptococcus dysgalactiae* subsp. *dysgalactiae* [1]. SDSE strains, particularly those isolated from humans, can express various carbohydrates, especially group C and G antigens, which are rhamnose-rich polysaccharides anchored to the bacterial peptidoglycan layer [2]. SDSE is closely related to *Streptococcus pyogenes* [3,4], a well-known pathogen responsible for numerous human infections such as pharyngitis, skin and soft tissue infections, bacteraemia, pneumonia and myositis. Additionally, these bacteria can also cause severe invasive infections such as necrotizing fasciitis (a rapidly spreading infection of the fascia, causing tissue death) and toxic shock syndrome – conditions commonly associated with *S. pyogenes* [3].

The genomes of SDSE and *S. pyogenes* exhibit extensive overlap, and both species share several virulence factors [3,4]. The genomic similarity is linked to their ability to cause a common wide spectrum of clinically similar diseases, including severe forms of illnesses [3]. In recent years, SDSE has garnered significant attention due to a noticeable increase in cases of invasive disseminated infections [5,9].

Implementing efficient, rapid and cost-effective techniques for bacterial clonal identification is crucial, as specific bacterial clones can exhibit distinct virulence and resistance profiles. In this context, matrix-assisted laser desorption/ionization-time of flight MS (MALDI-TOF MS) has become a prominent tool for pathogen identification, particularly in clinical microbiology [10].

MALDI-TOF MS offers several advantages, including speed, as it provides rapid identification of micro-organisms, typically within minutes to a few hours; simplicity, requiring minimal sample preparation compared to traditional culture methods; and cost-effectiveness, since although the initial investment may be high, the per-sample cost is relatively low, making it a valuable tool for routine microbiological practices [11,13].

PFGE is an important technique for investigating closely related outbreak scenarios in both hospital and community settings involving bacteria. However, it is time-consuming, expensive and requires specialized staff for running and analysing results. Additionally, variability in results between laboratories can make comparisons challenging [14,15]. Multilocus sequence typing (MLST) classifies bacterial strains based on the sequences of housekeeping gene sequences and has been applied to SDSE to differentiate strains and study epidemiology (https://pubmlst.org/). However, MLST is labour-intensive, time-consuming and may lack the resolution to distinguish bacterial strains. Whole-genome sequencing (WGS) is the most accurate method for assessing clonality, providing high-resolution data that allow for precise strain differentiation and evolutionary insights. Nevertheless, its high cost, longer turnaround time and the need for bioinformatics expertise limit its routine application in surveillance and clinical settings [15]. In this study, we evaluated the ability of MALDI-TOF MS to differentiate SDSE strains based on their clonality, as classified by PFGE band patterns.

## Methods

In this study, we investigated 115 SDSE strains previously characterized by Silva *et al*. [16] based on their PFGE band patterns: 66 strains corresponded to pulsotypes within PFGE pattern A (Table S1, available in the online Supplementary Material), 31 to pulsotypes of pattern B (Table S2) and 18 to sporadic pulsotypes (non-A/non-B) designated as PFGE patterns C to O (Table S3).

The sample preparation followed the methodology proposed by Pinto *et al*. [17], which has been successfully applied in their research on *Streptococcus pneumoniae*. Five colonies, obtained from 5% sheep blood Agar Base (Becton, Dickinson and Company, Franklin Lakes, NJ, USA), were resuspended in 5 µl of 70% formic acid (Tedia, Cincinnati, OH, USA) and mixed for 10 s. The same volume of high-performance liquid chromatography (HPLC)-grade acetonitrile (Tedia) was then added, followed by gentle homogenization. The mixture was centrifuged at 1,960 ***g*** for 3 min, and 1 µl of the supernatant was spotted onto a polished steel MSP 96 target plate (Bruker Daltonics, Bremen, Germany). After air-drying, each spot was overlaid with 1 µl of CHCA matrix (α-cyano-4-hydroxycinnamic acid; Bruker Daltonics). After preparation, the samples were analysed using a Microflex LT mass spectrometer (Bruker Daltonics) to generate spectra in the 2,000–20,000 mass-to-charge ratio (m/z) range employing the instrument’s default settings. These included a laser frequency of 60 Hz, lens voltage of 6 kV and ion source voltages of 2.0 and 1.8 kV.

The spectra were normalized using BioNumerics software v7.6 (Applied Maths, Ghent, Belgium) with default parameters. A running rolling disc algorithm was applied for baseline subtraction, and peak detection and matching were carried out in BioNumerics using a signal-to-noise ratio of 10 and a tolerance of ±0.002 m/z, following the methodology described by Pinto *et al*. [17]. BioNumerics was also used to align and compare the spectra of the strains with PFGE patterns A and B. The detected picks were exported into a spreadsheet, and manual inspection was performed to identify biomarkers uniquely present in PFGE pattern A or B. This combined approach allowed the selection of a set of specific biomarkers capable of clearly discriminating the predominant PFGE patterns A and B (Tables S1, S2 and S3).

To confirm whether the two main SDSE clusters classified by PFGE were also distinct at the phylogenomic level, WGS was performed on 20 randomly selected representative strains from PFGE patterns A and B, with selection carried out using the Excel RAND() function. Genomic DNA was obtained using the Wizard^®^ Genomic DNA Purification Kit (Promega, Madison, WI, USA). Libraries were prepared following the Nextera^®^ DNA Flex Library Prep Kit protocol (Illumina, San Diego, CA, USA), and paired-end sequencing was performed on an Illumina NextSeq2000 platform using a 300-cycle paired-end protocol. Raw sequencing reads were quality-trimmed with Trimmomatic v.0.39.2 (accessed at https://github.com/timflutre/trimmomatic) with a sliding window cut-off of Q15. Trimmed reads were *de novo* assembled into contigs using Unicycler v.0.4.8 (accessed at https://github.com/rrwick/Unicycler), and assemblies were quality-checked using QUAST v.5.0.2 (accessed at https://github.com/ablab/quast). The genome sequences are available in the National Center for Biotechnology Information (NCBI) database (accessed at https://www.ncbi.nlm.nih.gov/; BioProject PRJNA1278724; Table S4).

Molecular typing of the sequenced genomes was performed using the MLST tool (accessed at https://github.com/tseemann/mlst), based largely on *S. pyogenes*, targeting the housekeeping genes *gki*, *gtr*, *murI*, *mutS*, *recP*, *xpt* and *atoB*. Strains that could not be assigned a sequence type (ST) using this method were subsequently submitted to the PubMLST platform (accessed at https://pubmlst.org) for ST determination. *emm*-Typing was performed using emmtyper v0.2.0 (accessed at https://github.com/MDU-PHL/emmtyper).

A core genome tree was constructed using the 20 selected SDSE strains, with two *S. pyogenes* sequences (strain 37–97, GenBank accession no. CP041408.1; strain MGAS10750, GenBank accession no. CP000262.1) included as outgroups to root the tree. Genome annotation was performed using Prokka v1.14.6, and core genome alignments were generated with Roary v3.13.0, employing multiple alignment using fast fourier transform (MAFFT) and including only genes present in ≥99% of the genomes to ensure high-quality alignments. Prokka and Roary are available at https://github.com/tseemann/prokka and https://github.com/sanger-pathogens/roary, respectively. A maximum likelihood tree was inferred using RAxML Next Generation v.1.2.2 (https://github.com/amkozlov/raxml-ng) with the GTR+GAMMA substitution model and 100 bootstrap replicates. The Interactive Tree of Life v7 (accessed at https://itol.embl.de/) was used for tree visualization and metadata annotation.

Statistical analysis to assess the discriminatory power of the biomarkers in relation to their cladistic distribution on the phylogenomic tree was conducted using MedCalc software (accessed at https://www.medcalc.org/calc/diagnoStic_test.Php).

## Results

**Fig. 1.**
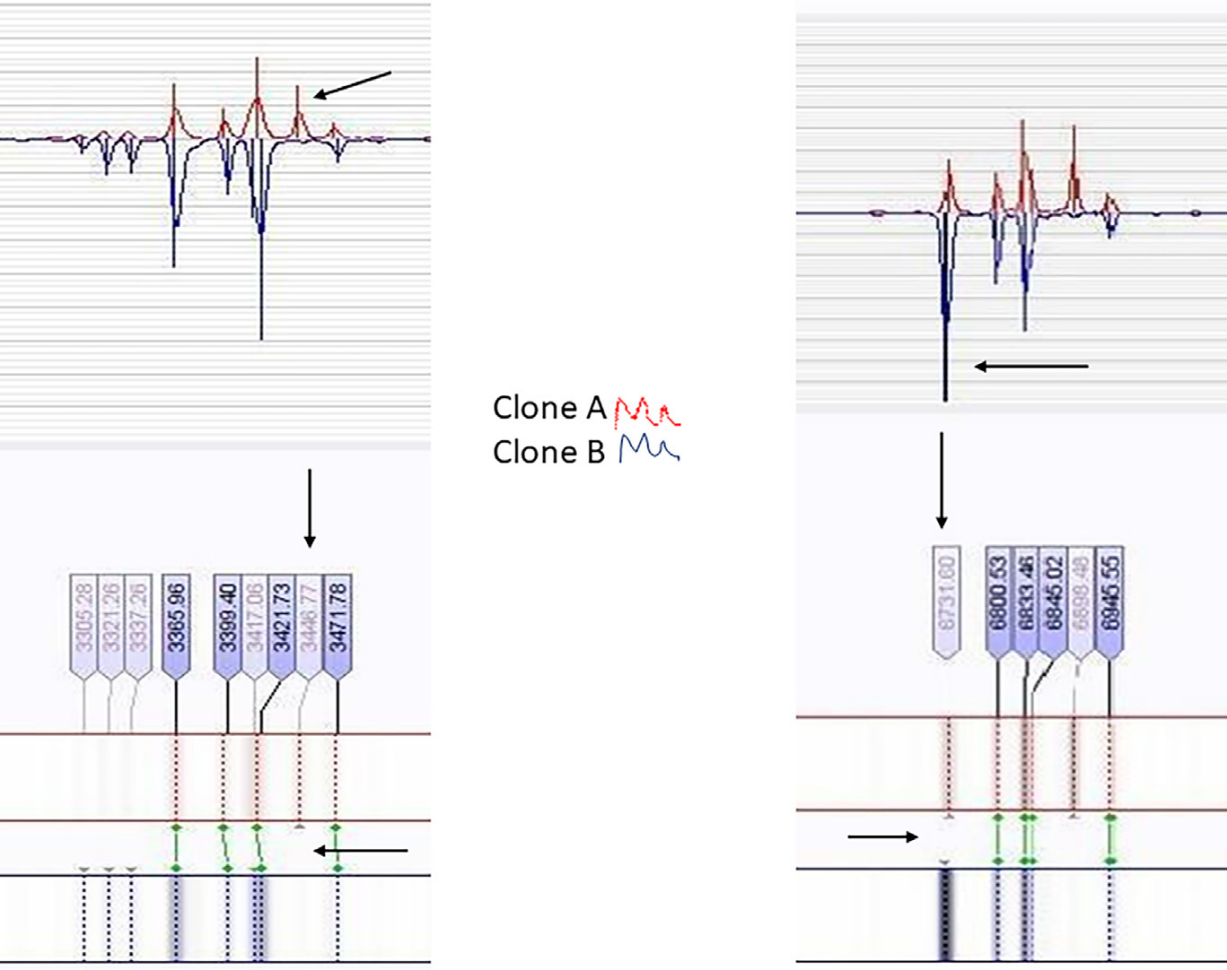
Spectra displaying the specific biomarkers identified in SDSE strains classified as PFGE patterns A and B by Silva *et al*. [16]. Arrows indicate absent connections (green line), highlighting the differences between the spectra of clone A and clone B, and vice versa.

The spectra generated for each strain contained an average of 93 peaks. For subsequent analysis, only peaks displaying intensity values exceeding 1,000 arbitrary units were considered. Remarkably, all strains exhibiting PFGE pattern A pulsotypes (*n*=66) displayed a distinct biomarker at 3446.77 m/z, which was consistently absent in strains corresponding to PFGE pattern B pulsotypes. In contrast, all pattern B strains (*n*=31) displayed a specific biomarker at 6731.60 m/z. Additionally, all 18 strains exhibiting sporadic PFGE pulsotypes (non-A/non-B) tested negative for both A- and B-specific biomarkers (Fig. 1 and Table 1).

**Table 1.** Specific biomarkers selected for distinguishing PFGE patterns of SDSE

PFGE* pattern	Biomarker (m/z)**	
	3446.77	6731.60
A	+†	−†
B	−	+
Non-A/non-B‡	−	−

*Previously identified by PFGE by Silva *et al*. [16].

†+, Presence of the biomarker; −, absence of the biomarker.

‡Strains displaying sporadic PFGE pulsotypes.

**m/z, mass-to-charge ratio.

To validate these findings, we conducted a blind test on a randomly selected set of strains comprising 15 PFGE pattern A strains, 15 pattern B strains and 5 non-A/non-B strains. All strains were accurately assigned to their respective clone types based on the detection of specific biomarkers. Notably, the biomarkers identified in clones A (3446.77 m/z) and B (6731.60 m/z) were absent in all sporadic PFGE strains tested.

**Fig. 2.**
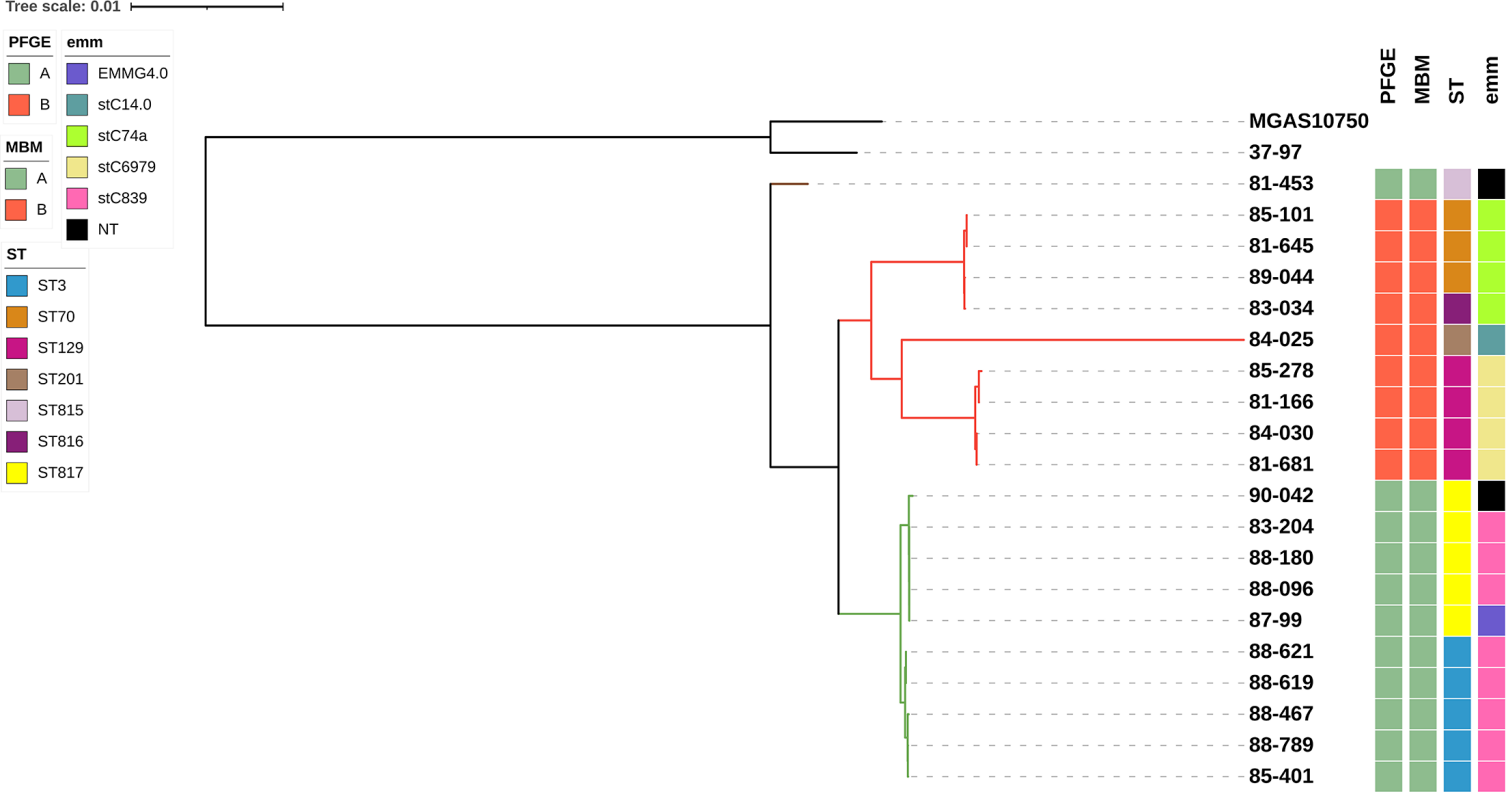
Phylogenomic analysis and identification of genomic biomarkers for PFGE A and B strains. The ML tree of 20 SDSE strains representing PFGE patterns A and B, alongside two outgroup strains (*S. pyogenes* strain 37–97, GenBank accession no. CP041408.1; strain MGAS10750, GenBank accession no. CP000262.1). Strains exhibiting PFGE patterns A and B are represented by green and red squares, respectively. The MBM at 3446.77 m/z, specific for PFGE A strains, is labelled in green, while the 6731.60 m/z biomarker, unique for PFGE B strains, is labelled in red. Most PFGE-A strains clustered within the same green clade (green-coloured branches), with the sole exception of strain 81–453, while all PFGE B strains consistently grouped within the red clade (red-coloured branches). ST, defined by MLST, and *emm* types are represented by coloured squares. MBM, MALDI-TOF MS biomarker; ML, maximum likelihood; NT, non-tippable.

The phylogenomic tree structure resolved PFGE A and B strains into two distinct main clades (highlighted in green and red, respectively). The only exception was the strain 81–453, previously classified as exhibiting a PFGE A pulsotype and displaying the MALDI-TOF MS peak at 3446.77 m/z, which fell outside the green clade in this tree. However, it did not cluster within the red clade either; instead, it fell closer to the common ancestor of both green and red genome clades, exhibiting genomic features shared by both clusters. Considering the phylogenomic tree as the reference, the biomarkers demonstrated a sensitivity of 100% [95% confidence interval (CI): 66.4–100%], specificity of 91.7% (95% CI: 61.5–99.8%), positive predictive value of 90.0% (95% CI: 58.0–98.3%), negative predictive value of 100% (95% CI: 71.5–100%) and overall accuracy of 95.2% (95% CI: 76.2–99.9%). MLST profiles varied both between and within the two clusters, whereas the *emm* type was more uniform within the green cluster. Indeed, strains in the green clade were less diverse, all belonging to clonal complex (CC) 3 (CC3; ST3 and ST817), and were more closely related, as reflected by the phylogenomic tree, compared with strains from the red clade, which included mostly ST70 and ST129 belonging to different CCs (Fig. 2 and Table S4). Additionally, this study includes the first report of ST815, ST816 and ST817 within the current MLST framework.

Although MALDI-TOF MS successfully distinguished the two main phylogenetic clusters of SDSE, our attempt to identify corresponding genomic biomarkers based on the presence or absence of specific ORFs was unsuccessful. This discrepancy likely reflects the nature of the MALDI-TOF signal. Variations in the MALDI-TOF spectra can arise from subtle amino acid substitutions, post-translational modifications or differential protein expression, none of which are necessarily captured by simple presence/absence analyses of ORFs. Additionally, genes encoding these proteins may reside in genomic regions that are difficult to assemble, such as repetitive sequences or plasmids [18].

## Discussion

We demonstrated the potential of MALDI-TOF MS to discriminate between PFGE-defined patterns among SDSE strains. The analysed strains had been previously grouped into two main clusters (A and B) based on PFGE band-pattern analysis using the GelCompar II software (Applied Maths, Sint-Martens-Latem, Belgium) [16]. The MALDI-TOF MS system accurately differentiated these clusters, detecting specific peaks at 3446.77 m/z for PFGE pattern A and 6731.60 m/z for PFGE pattern B. Other studies have similarly explored the application of MALDI-TOF MS as a valuable tool in bacterial epidemiology. For instance, Pinto *et al*. [17] successfully employed a MALDI-TOF MS approach to identify distinct biomarkers for differentiating strains of *S. pneumoniae*. Additionally, Moura *et al*. [19] utilized the MALDI-TOF MS system to identify distinct biomarkers for different *emm* types of *S. pyogenes*. Studies involving other streptococci, such as group B *Streptococcus* (GBS), have also demonstrated the ability of MALDI-TOF MS to identify specific protein peaks to distinguish strains of ST 1 [20]. Huang *et al*. [21] further explored GBS strains and identified unique peak markers for ST10 (6250, 3125 and 6891 m/z) and ST17 strains (2956, 5912, 7735 and 5218 m/z). These findings reaffirm the significant potential of MALDI-TOF MS for molecular epidemiological monitoring through the use of biomarkers. Recently, the same research group expanded their protocol based on mass spectra obtained from MALDI-TOF MS to identify five CCs of GBS (CC10, CC12, CC17, CC19 and CC23) [22].

In our dataset, the molecular biomarker displayed perfect correspondence with PFGE types, discriminating pattern A from pattern B with 100% concordance. However, when mapped onto the whole-genome phylogeny, one isolate classified as PFGE-A (*n*=10) clustered outside the A clade (green), in a position closer to the most recent common ancestor of clades green and red. All PFGE-B strains (*n*=10), in contrast, were consistently recovered within the B clade (red). These findings underscore that, although the biomarkers robustly discriminate PFGE types, their phylogenetic signal is only partially congruent with the whole-genome clade structure. Nevertheless, when taking the phylogenomic tree as the reference, the biomarker achieved an overall accuracy of 95.2% (95% CI: 76.2–99.9%), highlighting its usefulness for rapid typing despite not fully capturing evolutionary relationships, likely due to ancestral polymorphisms or horizontal gene transfer events.

The presence of different MLST profiles among strains clustered within the same PFGE patterns suggests that PFGE classification is more conserved than the MLST scheme used for SDSE, which was adapted from *S. pyogenes* [23]. Thus, this MLST scheme may not adequately capture the genetic diversity and evolutionary features of SDSE, potentially hindering both epidemiological tracking and the understanding of resistance and virulence evolution. Although further studies are needed to clarify this issue, previous MLST analyses of SDSE isolates from Australia, Portugal and the USA reported a high degree of genetic diversity and revealed the occurrence of lateral gene transfer of housekeeping alleles [23,24].

While WGS is expected to become the method of choice for clonality studies in the future due to its precision and ability to provide insights into resistance, virulence and bacterial evolution, its routine application remains limited by cost and technical demands [15]. Ge *et al*. [11] estimated a cost of approximately One United States dollar and sixty-three cent (USD 1.63) per sample for microbial identification by MALDI-TOF MS, including equipment depreciation and consumables, whereas Brown *et al*. [25] reported that WGS costs at least USD 400 per isolate. These figures highlight the significant economic advantage of MALDI-TOF MS, particularly in high-demand or resource-limited settings, while maintaining operational simplicity and applicability for routine epidemiological surveillance. In this context, despite the homoplastic distribution of biomarker A relative to the phylogenomic clade, its high sensitivity and specificity support the use of MALDI-TOF MS biomarkers for SDSE clone tracking, with WGS reserved for confirmatory analysis.

The clinical significance of strains exhibiting PFGE patterns A and B lies not only in their predominance in both infection and colonization cases but also in their enhanced pathogenic potential. These strains displayed greater adhesion to and invasion of respiratory epithelial cells, harboured a higher number of virulence-associated genes and formed more robust biofilms compared to sporadic strains. Furthermore, they exhibited increased virulence in animal models [4,16]. Identifying and monitoring these high-risk clones is therefore crucial for understanding transmission dynamics, guiding targeted infection control measures and improving patient management in both hospital and community settings.

A limitation of our study is that the strains analysed were drawn from a convenience collection and may not necessarily represent epidemiologically linked cases, which could influence the generalizability of our findings regarding PFGE-defined clusters. Another limitation is that the chemical nature of the MALDI-TOF biomarkers could not be directly determined. Nevertheless, our findings underscore the potential of molecular markers identified by MALDI-TOF MS as an effective tool for tracing more virulent and widespread clones of SDSE involved in invasive and severe infections, offering a rapid and accurate approach to clonal identification. Overall, these results reinforce the significant potential of MALDI-TOF MS for molecular epidemiological monitoring of streptococci. For certain streptococcal species, including SDSE, *S. pyogenes*, *S. pneumoniae* and GBS, the MALDI-TOF MS system holds considerable promise for applications in bacterial epidemiology, particularly in tracking widespread clones. These findings highlight its utility not only for species identification but also for improving our understanding of bacterial diversity and the epidemiological relationships among different strains.

## Supplementary material

10.1099/jmm.0.002080 Uncited Supplementary Material 1.

## Abbreviations

CC: clonal complex
CI: confidence interval
GBS: group B *Streptococcus*
HPLC: high-performance liquid chromatography
MAFFT: multiple alignment using fast fourier transform
MALDI-TOF MS: matrix-assisted laser desorption/ionization-time of flight MS
MLST: multilocus sequence typing
m/z: mass-to-charge ratio
NCBI: National Center for Biotechnology Information
SDSE: *Streptococcus dysgalactiae* subsp. *equisimilis*
ST: sequence type
USD: United States dollar
WGS: whole-genome sequencing
